# Serious Workplace Violence Against Healthcare Providers in China Between 2004 and 2018

**DOI:** 10.3389/fpubh.2020.574765

**Published:** 2021-01-15

**Authors:** Jing Ma, Xi Chen, Qiongjuan Zheng, Yun Zhang, Zhi Ming, Dongxin Wang, Hua Wu, Haisen Ye, Xiaoxuan Zhou, Yunxuan Xu, Renjiao Li, Xia Sheng, Fangxiu Fan, Zuiwen Yang, Ting Luo, Yajun Lu, Ye Deng, Fen Yang, Chuntao Liu, Chunyu Liu, Xiaosong Li

**Affiliations:** ^1^Department of Child and Adolescent Psychiatry, School of Clinical Medicine, The Second People's Hospital of Hunan Province, Hunan University of Chinese Medicine, Changsha, China; ^2^Medical College of Northwest University for Nationalities, Lanzhou, China; ^3^Furong Forensic Center of The Second People's Hospital of Hunan Province, Yuhua, China; ^4^Department of Psychiatry, Department of Neuroscience and Physiology, SUNY Upstate Medical University, Syracuse, NY, United States; ^5^Hunan Provincial People's Hospital, Changsha, China

**Keywords:** serious workplace violence, healthcare, reasons, outcome, China

## Abstract

**Introduction:** Workplace violence (WPV) against healthcare providers has severe consequences and is underreported worldwide. The aim of this study was to present the features, causes, and outcomes of serious WPV against healthcare providers in China.

**Method:** We searched for serious WPV events reported online and analyzed information about time, location, people, methods, motivations, and outcomes related to the incident.

**Result:** Serious WPV reported online in China (*n* = 379) were mainly physical (97%) and often involved the use of weapons (34.5%). Doctors were victims in most instances (81.1%). Serious WPV mostly happened in cities (90.2%), teaching hospitals (87.4%), and tertiary hospitals (67.9%) and frequently in Emergency Department (ED), Obstetrics and Gynecology Department (OB-GYN), and pediatric departments; it was most prevalent in the months of June, May, and February. Rates of serious WPV increased dramatically in 2014 and decreased after 2015, with death (12.8%), severe injury (6%), and hospitalization (24.2%) being the major outcomes. A law protecting healthcare providers implemented in 2015 may have helped curb the violence.

**Conclusion:** Serious WPV in China may stem from poor patient–doctor relationships, overly stressed health providers in highly demanding hospitals, poorly educated/informed patients, insufficient legal protection, and poor communication. Furthering knowledge about WPV and working toward curtailing its presence in healthcare settings are crucial to increasing the safety and well-being of healthcare workers.

## Introduction

Workplace violence (WPV) refers to an individual's or group's socially unacceptable, aggressive (and sometimes destructive) behavior (1–3). WPV against healthcare workers is a global public health problem that has been underreported and largely ignored (4). World Health Organization (WHO) estimated that 8–38% of healthcare workers suffer from physical violence while working in 2019 (5). Many more are threatened or exposed to verbal aggression (6). The damage due to workplace violence translates into physical and mental harm to the health workers (7). The research literature shows that such violence leads to issues such as death (8), heart and brain disease (9), anxiety, depression (10), and posttraumatic stress disorder (PTSD) (11, 12). Workplace violence also translates to high costs for the organization where it takes place, both in the short and long term, and decreases quality of care provided to all patients (13, 14). In China, workplace violence in hospitals causes a lot of to change their majors and decreases the integrity of the healthcare provider–patient relationship (15).

The perpetrators who carry out violent behavior against healthcare workers vary with respect to their relationship to the worker: some are patients, some are patients' relatives, and others are neither (16). Research literature from Greece and Nepal has shown that nurses are more likely to be the victims of WPV than doctors (17, 18) and that verbal violence is more common than physical violence (6, 13, 19). However, a study in China showed that doctors are more frequently the victims compared to the nurses (20). Additionally, physical violence against doctors appears to be more common than physical violence against nurses in China (21). There are only a few studies on WPV in China (17, 19, 22); the prevalence of WPV varies from province to province (21, 23), from hospital to hospital (19, 24), and from department to department (25, 26). China is the only country in which prevalence of WPV by month has been studied; according to previous research, it is most common in July (20).

Many researchers have tried to determine the reasons behind WPV, which can vary as a result of different medical systems and national conditions. There is a lot of literature that explores the outcomes of WPV (27, 28). Of all the countries with research on the topic, we found that WPV in China leads to the most serious outcomes (8).

Serious WPV against healthcare workers, although less common than milder forms of violence, possibly gets more attention from mass media and the public. It shows the worst relationship between healthcare providers and patients and also reflects particularly negative living situations of healthcare providers in certain medical systems. It reveals the suffering and helplessness of patients, as well as the defects of certain medical and legal systems. Serious WPV usually happens suddenly, which makes research on the topic hard to carry out through routine methods like checklists and interviews. Studying mass media reports may therefore currently be the best way to study serious WPV.

As far as we know, there have only been two studies about serious WPV against healthcare workers in China (with a few more studies focusing on less serious WPV) (8, 20). One of these articles examines the changes in prevalence and features of serious WPV against doctors and nurses in China, as reported online from 2000 to 2015 (8), but it did not study the reasons for and outcomes of serious WPV. This article will present the newest changes in, features of, reasons for, and outcomes of serious WPV trends against healthcare providers in China from 2004 through 2018 based on online reports.

## Methods

The research data examined in this article came from online reports about workplace violence against healthcare workers in hospitals from January 2004 to December 2018. Baidu, Sogou, Souhu, and Lilac Garden were used as search engines, and “ShangYi” (do harm to doctors), “Yi Yuan,” and “Bao Li” (hospital and violence), “Yi Nao” (medical harassment), “Da Yi Sheng” (beating doctors), “Da Hu Shi” (beating nurses), “Yi Huan Chong Tu” (healthcare provider-patient disputes), and “Bao Li Shang Yi” (healthcare workers' injury by workplace violence) were used as search words for finding news and reports online.

Relevant online information was screened, and secondary materials were excluded. We read the reports and collected the following information about the violence: causes, time (year, month), region (province, city, county, town), hospital (name, public/private, level of the hospital if public), department, types of violence (verbal, physical, or both), identity of victims (doctor, nurse, other staff member), identity of perpetrators (patient, relative of the patient, other person), and outcomes of the events [death, injury, type of injury, admission to inpatient department (IPD) or not]. We asked a coroner to read the outcome information that we collected and to determine how serious the injuries were (severe injury, minor wound, or slight bodily injury).

This study has been approved by the Ethics Committee of the Hunan Provincial Brain Hospital, ethics approval number 59.

SPSS17.0 was used to input data and to do statistical analyses. We calculated frequency and proportion of serious WPV with regards to location (province, city, county, town, hospital, and department), time (monthly and yearly changes), outcomes, reasons for violence (losing control of emotions, dissatisfaction and high expectations for treatment outcomes, unreasonable request for procedures), features of violent behavior, and identity of perpetrators and victims. The incidence of serious WPV in 2014 showed an increase compared to previous years. The difference in WPV rates across years was explored using chi-square test.

## Results

### Sample Size

There were 379 violent events reported from January 2004 to December 2018. Some information was not included in the reports, which led to missing values. However, there was complete information for province, year, and name of hospital. The number of reports that included information for the remaining fields are as follows: department, 219; month, 378; day, 371; city, 368; hospital level, 258; teaching hospital or not, 364; identity of victim, 370; types of violence, 370; reasons:, 372; with weapon or not, 365; identity of perpetrator, 331; and outcomes, 265.

### Identity of Victims and Perpetrators and Features of Violent Behavior

Doctors were victims in 300 events (81.1%), nurses were victims in 134 events (36.2%), and both nurses and doctors were injured in 64 events (17.3%). There were 30 events in which other persons (security guards, policemen, etc.) were injured, too.

Most common perpetrators were relatives of the patients (190 events, 57.4%), followed by patients themselves (132 events, 39.9%), and in some events, both patients and their relatives carried out the violent behavior (12 events, 3.6%). There were 28 acts of violence (8.4%) committed by nonrelatives of patients.

The reported violent events included physical violence (beating, slapping of the face, stabbing with knife, hitting with bricks/chairs, forcing victims to kneel, kidnapping, stalking, etc.) 97% of the time (*n* = 359). Verbal violence (insulting, cursing, swearing, shouting, threatening, intimidating, etc.) occurred in 21.1% of the events (*n* = 78). Almost one-fifth (*n* = 67, 18.1%) of the events included both physical and verbal violence, and 34.5% of perpetrators used a weapon (knife, brick, stick, table, stairs, etc.).

### Location: Province, City, County, Town, Hospital, and Department

There are 31 provinces and 4 municipalities in China, and none of them were free from workplace violence between 2004 and 2018, although the frequency of incidence varied from province to province. The five provinces/municipalities with the most WPV and the percentage of the total incidents that occurred in each are as followings: Guangdong, 52 (13.7%); Hunan, 30 (7.9%); Jiangsu, 25 (6.6%); Beijing, 22 (5.8%); and Guangxi, 21 (5.5%).

Most of the workplace violence in hospitals happened in cities (332, 90.2%), while only 36 events (9.8%) happened in counties and towns. Most of the workplace violence happened in tertiary hospitals (243, 67.9%), which are the highest-ranking hospitals in the system, and only rarely in first-level hospitals (10, 2.8%) or private hospitals (8, 2.2%). The remainder either happened in secondary hospitals (72, 20.1%) or other public hospitals whose level was not mentioned (25, 7.0%). Remarkably, of all the hospitals that reported violent workplace incidents, teaching hospitals accounted for 87.4% (318) while non-teaching hospitals accounted for 12.6% (46).

The top three departments with the highest rates of serious WPV were Emergency Departments (74, 33.8%), Obstetrics–Gynecology Departments (26, 11.9%), and pediatrics departments (20, 9.1%). In total, 18.3% of events happened in a department related to internal medicine (*n* = 40). A total of 20.1% of events happened in departments related to surgery not associated with obstetrics–gynecology (*n* = 44).

### Time: Year and Month

**Figure 1:**
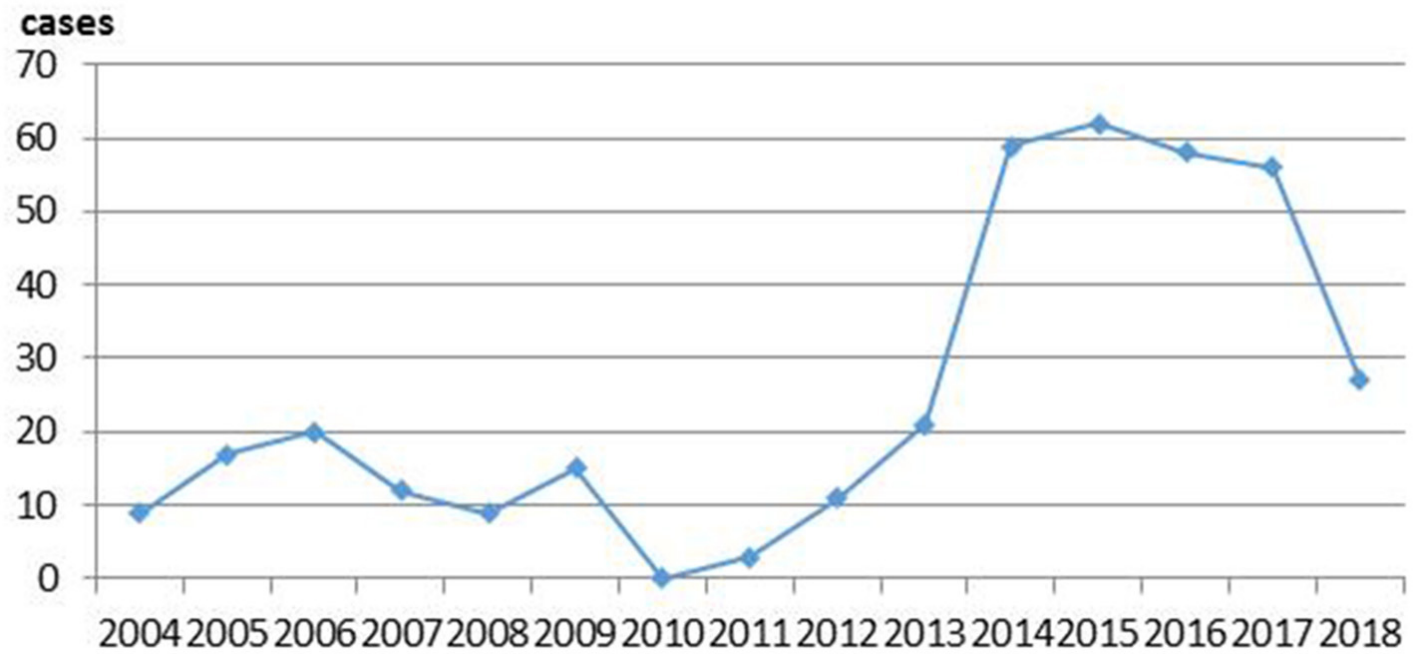
The frequency of serious workplace violence (WPV) between 2004 and 2018.

From 2004 to 2013, the incidence rate of WPV fluctuated. The incidence increased significantly over the year during 2014 and peaked in 2015, then decreased gradually in the following years. By the end of the timeframe of interest, rates had decreased to the lowest levels between 20014 and 2018, which were almost as low as the rates in 2013 (see Figure 1).

As shown in Table 1, there was no difference between the risk of serious WPV occurring in 2012 (“a”) vs. 2013 (“a”), but there was a statistically significant difference (*P* < 0.05) between the risk of serious WPV occurring in 2013 (“a”) vs. 2014 (“b”), and in 2012 (“a”) vs. 2014 (“b”).

**Table 1:** The incidence and comparison of serious WPV in 2012, 2013, and 2014.

			**Year**			**Total**
			**2012**	**2013**	**2014**	
Whether serious WPV happened this year	Yes	Count	10^a^	15^a^	39^b^	64
		Percentage (%)	15.60%	23.40%	60.90%	100.00%
	No	Count	255^a^	250^a^	226^b^	731
		Percentage (%)	34.90%	34.20%	30.90%	100.00%
Total		Count	265	265	265	265
		Percentage (%)	33.30%	33.30%	33.30%	33.30%
Pearson chi-square		Value	24.504			
		*P*	<0.001			

*Each subscript letter denotes a subset of year categories whose column proportions do not differ significantly from each other at the 0.05 level*.

**Figure 2:**
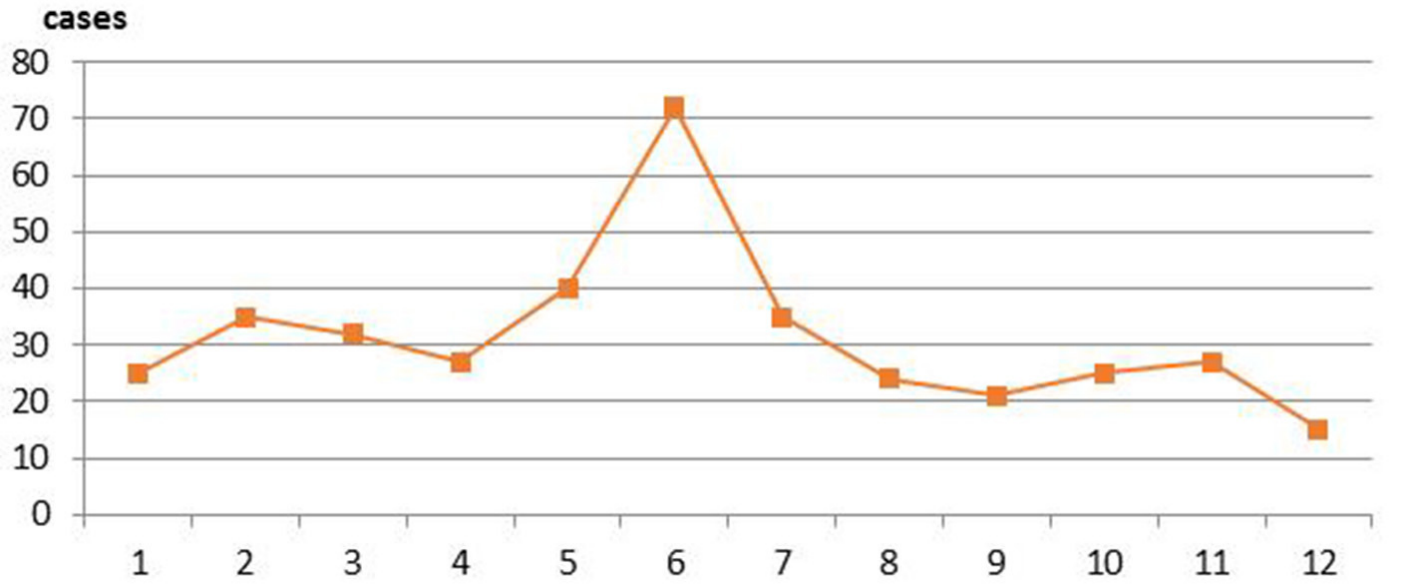
Month distribution of serious workplace violence (WPV) between 2004 and 2018.

The 5 months with most occurrences of serious WPV were June (72, 19%), May (40, 10.6%), July (35, 9.3%), and February (35, 9.3%) (see Figure 2).

### Outcome of Violent Events

Two hundred sixty-five reports (69.9% of the total sample) included information about the outcome of the injured persons. Out of these 265 reports, 171 had detailed description of injury severity and target body parts.

The severity of injuries was as follows: death (34, 12.8%); severe injury, such as pierced heart, paralysis of both lower limbs, decapitated arm, or intestinal perforation (16, 6.0%); minor wound, such as intracranial hemorrhage, orbital fracture, concussion, miscarriage, second-/third-degree burn, tendon rupture, or lung contusion (70, 26.4%); and slight bodily injury, such as light closed encephalon injury, threatened miscarriage, soft tissue contusion, nose bleeding, head trauma, facial blood stasis, or waist injury (82, 30.9%). It is worth mentioning the injuries that were classified as “minor” were categorized according to a forensic standard in China and that many of these injuries would not be considered minor by most people.

Moreover, the injured persons suffered head and face injuries in 102 events (38.5%) and trunk injuries in 33 events (12.4%), while limb injuries accounted for 6.1% (16) of injuries; notably, many people suffered multiple injuries in 1 event; 64 events (24.2%) led to hospitalization.

### Reasons for Violent Behavior

The reasons behind the perpetrators' acts of violence in the hospital were as follows: refusing to accept the death of the patient (12.6%, 47), being dissatisfied with the treatment outcomes (10.5%, 39), thinking that the emergency treatment is not effective (7.3%, 27), wanting to get treatment as soon as possible without following medical procedures (6.5%, 24), being drunk (3.5%, 13), having a suspected mental disorder (3.2%, 12), believing that adverse effects of treatment were due to clinical operations (3.0%, 11), failure of operation (such as puncturing), leading to the need for a second operation (2.7%, 10), having a diagnosed mental disorder (1.3%, 5), asking staff for particular treatment and arrangements but being refused (1.08%, 4), and unspecified reasons (20.2%, 75).

## Discussion

The purpose of this study was to reveal the features of serious WPV reported online against healthcare providers in China. Our results convey insights into the people involved in, the times of, the locations of, as well as the methods used for and the outcomes of serious WPV in Chinese hospitals. The major findings were as follows. (1) The vast majority of cases of serious WPV reported online were physical in nature (97%) and were often committed with weapons (34.5%). Doctors were more exposed to serious WPV than nurses. (2) Guangdong, Hunan, Jiangsu, Beijing, and Guangxi had the highest rates of serious WPV; Qinghai, Hainan, Ningxia, Neimeng, Taiwan, and Shanxi had the lowest prevalence rates. (3) Serious WPV mostly happens in cities (90.2%) and usually occurs in tertiary hospitals (67.9%), especially teaching hospitals, which account for 87.4% of serious WPV events in tertiary hospitals. (4) The three departments making up the highest proportion of serious WPV incidents were Emergency Department (ED), Obstetrics and Gynecology Department (OB-GYN), and pediatrics. (5) Serious WPV increased dramatically in 2014 and decreased gradually after 2015. The 3 months during which serious WPV occurred most frequently between 2004 and 2018 were June, May, and February. (6) The major reasons behind serious WPV were losing control of emotions, dissatisfaction with and high expectations for treatment outcomes, and unreasonable requests for procedures.

### Who and How

Our study found that the vast majority of cases of serious WPV reported online were physical in nature (97%) and were often committed with weapons (34.5%). Such a high occurrence of weapon use suggests extreme conflict between patients and healthcare providers. Our study also found that doctors were more exposed to serious WPV than nurses. Several previous studies showed that nurses were more exposed to verbal violence than doctors (6, 17, 29–31) and that doctors were more often the victims of physical workplace violence (32, 33). These results indicate that the most serious WPV may result from more major issues such as those related to diagnosis and treatment—which are primarily linked to doctors—rather than from smaller issues related to nurse–patient interactions. Perpetrators are more often relatives of patients than they are patients themselves. This finding has been reported cross-culturally (34, 35). This may be because patients are sometimes unable to move or to argue or fight due to medical conditions and age. Relatives may express themselves through violence as a result of anger, worry, dissatisfaction, or financial intentions (claim for compensation).

### Where

Guangdong, Hunan, Jiangsu, Beijing, and Guangxi had the highest rates of serious WPV; Qinghai, Hainan, Ningxia, Neimeng, Taiwan, and Shanxi had the lowest prevalence rates. Previous similar research in China reached similar conclusions, with the greatest prevalence occurring in Guangdong, Jiangsu, Sichuan, and Zhejiang and the lowest rates occurring in Gansu, Ningxia, Tianjin, Shanxi, and Taiwan (8). We searched the populations and gross domestic products (GDPs) of the above provinces on the National Bureau of Statistics of China's website (http://data.stats.gov.cn/search.htm) and found that the provinces with the top number of serious WPV incidents had high GDPs or large populations. Most provinces with low prevalence of WPV had among the lowest GDPs or among the smallest populations in China. We speculate that economically developed provinces attracting millions of migrant workers every year—and thus adding to the already overloaded burden of the health providers by local residents—has led to a higher frequency of serious WPV. Such a great need for medical attention may strain medical staff resources and thus result in worse patient–doctor relationships, contributing to the prevalence of serious WPV. The relationship between these socioeconomic variables and serious WPV is complicated and requires further research.

This study found that serious WPV mostly happens in cities (90.2%) and usually occurs in tertiary hospitals (67.9%)—especially teaching hospitals—which account for 87.4% of serious WPV events in tertiary hospitals. The finding regarding teaching hospitals differs from the conclusions of the study of Chen et al., which showed that the incidence of WPV in teaching hospitals was lower than the incidence of WPV in regional hospitals in China and was similar to incidences in developed countries (36). Difference in methods and regions of interest may account for the discrepancy between these findings. Some studies (8, 21, 37), such as the report of Yen et al. on Heilongjiang, have shown that Chinese tertiary hospitals usually have higher rates of WPV than hospitals in rural areas or small towns (37). The current study found similar results. It is worth mentioning that tertiary hospitals in cities of China usually have the best equipment and the best doctors, where patients with comparatively severe, challenging diseases usually seek help. That means that, at these kinds of hospitals, the death toll per year can be expected to be higher, increasing motivation behind serious WPV. Furthermore, almost every doctor in teaching hospitals faces great pressure to do research and publish articles in order to get a promotion, which forces them to reduce clinical hours. Routine service in the inpatient units of teaching hospitals is mostly performed by resident trainees, postgraduate students, and further educational doctors. These doctors have less experience interacting with patients, as well as fewer medical skills and abilities, which may raise their likelihood of getting into medical disputes (38).

According to our findings, the three departments making up the highest proportion of serious WPV incidents were ED, OB-GYN, and pediatrics. Emergency departments have been previously described as being at high risk for violent incidents (8, 20, 25, 33, 34, 39, 40), a finding that was corroborated by the current study. Samir et al. found that 86.1% of nurses in OB-GYN departments had been exposed to WPV (35). Li found that Chinese healthcare providers in children's hospitals experienced violence commonly and that 68.6% of staff members had experienced at least one WPV incident in the past year (34). Ferri et al. found that the top 3 departments for WPV were psychiatry (86%), emergency (71%), and geriatric wards (57%) (30). However, the study of Min et al. from China showed that the frequency of WPV in OB-GYN (9) and pediatric departments (7) were not higher than in other internal medicine and surgical departments (8). We think that the high number of incidents reported in OB-GYN departments and pediatrics may have to do with the dramatic increase in maternal and child care hospitals (primarily pediatrics and OB-GYN), as well as children's hospitals in China between 2014 and 2018. Serious WPV occurring in these child-related hospitals increases the counts in those related departments.

### When

We found that serious WPV increased dramatically in 2014 and decreased gradually after 2015. This shift may be the result of an article (article 31, page 11) added to the Criminal Law of the People's Republic of China (ninth revision) in 2015. The article reads: “Where people are gathered to disturb public order to such a serious extent that work in general, production, business operation, teaching or scientific research cannot go on and heavy losses are caused, the ringleaders shall be sentenced to fixed-term imprisonment of not <3 years but not more than 7 years; the active participants shall be sentenced to fixed-term imprisonment of not more than 3 years, criminal detention, public surveillance or deprivation of political rights.”

The 3 months during which serious WPV occurred most frequently between 2004 and 2018 were June, May, and February. February is usually the month in which Spring Festival occurs. During Spring Festival, there is a shortage of staff members in hospitals, which may heighten risk of medical disputes. A report published in China by Yuqing et al. found that the top 3 months for WPV were May, June, and July (20). No research thus far has revealed the reasons behind the inordinately high amounts of serious WPV in June and May. This may be a good area of research for future study.

### Outcome

Serious WPV has very severe consequences. We are shocked by the rate of death (12.8%), severe injury (6%), and hospitalization (24.2%) that has resulted from serious WPV. Previous research in developed countries has reported that WPV has more frequently resulted in nonphysical harm (9–12, 41). On the contrary, a research in China—including this study—has found that physical harm is more common; these instances of physical violence have sometimes led to death (8).

### Reasons

We separated the reasons we found for serious WPV into three categories:

(1) Losing control of emotions, including: “being drunk” and “having a diagnosed or suspected mental disorder.” Previously, Bataille et al. found that alcohol abuse is one of the most common triggers of WPV in ED (42); a lot of other research has similarly found that drunkenness and mental disorders are often associated with physical violence against healthcare providers (30, 43–47).
(2) Dissatisfaction and high expectations for therapeutic outcome, including “failed clinical operation (like puncturing),” “operating for the second time,” “thinking the emergency treatment is not effective,” “believing that severe adverse effects of treatment were due to clinical operations,” “refusing to accept the death of the patient,” and “being dissatisfied with the treatment outcomes.” The dissatisfaction of treatment outcome was due to two reasons: either actual poor quality of medical care or unreasonable expectations leading to dissatisfaction in the face of normal medical failures or flaws. Previous research showed similar results pertaining to ineffective treatment and high expectations related to WPV (48), but the level of physical harm we reported was more serious. We speculate that these intense conflicts in China stem, in part, from negative healthcare provider–patient relationships and a lack of relevant legal measures. Both of these issues may be consequences of flaws in the medical system. This also may be a good area of research for future study.
(3) Unreasonable requests for procedures, including “asking the professional staff to give treatment and arrangements as requested but being refused” and “wanting to get emergency treatment as soon as possible without following medical procedures.” Alkorashy et al. found that misunderstandings and long waits for service are factors that contribute to WPV (49). Inadequate professional resources and poor communication between healthcare providers and patients may also sometimes be reasons behind unreasonable requests for procedures (33).

## Limitation

The main limitation of this study was that it was based on online reports, whose integrity and authenticity were influenced by factors such as government regulations, areas where reports were made, the interests of public media and internet companies, the professional ethics of the journalists responsible for the reports, and the validity of the resources. There is a chance that some incidents that occurred in rural areas and underdeveloped regions were not reported and thus not included in this study, which could bias some of the analyses.

## Conclusion

The current findings reflect a bleak healthcare setting in China, dangerous conditions for healthcare workers, and poor doctor–patient relationships, which may, in large part, be due to problems with the Chinese medical system, including overstressed health providers in the highly demanding hospitals, poorly educated/informed patients, insufficient legal protection, and poor communication between care providers and patients. Workplace violence against healthcare workers in China poses a serious threat to the well-being of doctors, nurses, and other providers; it may also be particularly distressing to more junior providers at the beginning of their careers, given the relatively high rate of WPV in teaching hospitals. We strongly believe that public education should be improved to reduce patients' unreasonable expectations. Furthermore, better allocation of medical resources and more legal action against serious WPV could reduce serious workplace violence.

## Data Availability Statement

The data analyzed in this study is subject to the following licenses/restrictions: If necessary, the data can be obtained by contacting the corresponding author. Requests to access these datasets should be directed to Chunyu Liu, liuch@upstate.edu.

## Author Contributions

JM, XC, QZ, YZ, ZM, DW, HW, HY, XZ, YX, RL, XS, FF, ZY, TL, YL, YD, FY, and ChuntL contributed to data acquisition. ChunyL and XL came up with the idea of doing this research and are responsible for the whole work. JM, XC, and QZ participated in data analysis and writing and revision of the article. All authors read and approved the final version of the manuscript.

## Conflict of Interest

The authors declare that the research was conducted in the absence of any commercial or financial relationships that could be construed as a potential conflict of interest.

## Abbreviations

WPV: workplace violence
WHO: World Health Organization
PTSD: posttraumatic stress disorder
ED: Emergency Department
OB-GYN: Obstetrics and Gynecology Department.
